# Identification of genetic factors underlying persistent pulmonary hypertension of newborns in a cohort of Chinese neonates

**DOI:** 10.1186/s12931-019-1148-1

**Published:** 2019-08-05

**Authors:** Xu Liu, Mei Mei, Xiang Chen, Yulan Lu, Xinran Dong, Liyuan Hu, Xiaojing Hu, Guoqiang Cheng, Yun Cao, Lin Yang, Wenhao Zhou

**Affiliations:** 1 Clinical Genetic Center, Children’s Hospital of Fudan University, 399 Wanyuan Road, Shanghai, 201102 China; 2 Department of Neonatology, Children’s Hospital of Fudan University, 399 Wanyuan Road, Shanghai, 201102 China; 3 Department of Pulmonology, Children’s Hospital of Fudan University, 399 Wanyuan Road, Shanghai, 201102 China; 4 Key Laboratory of Birth Defects, Children’s Hospital of Fudan University, 399 Wanyuan Road, Shanghai, 201102 China

**Keywords:** Persistent pulmonary hypertension of the newborn, Exome sequencing, Chinese, PAH genes, Genetic polymorphisms, Early diagnosis

## Abstract

**Background:**

Persistent pulmonary hypertension of the newborn (PPHN) is a severe clinical problem among neonatal intensive care unit (NICU) patients. The genetic pathogenesis of PPHN is unclear. Only a few genetic polymorphisms have been identified in infants with PPHN. Our study aimed to investigate the potential genetic etiology of PPHN.

**Methods:**

This study recruited PPHN patients admitted to the NICU of the Children’s Hospital of Fudan University from Jan 2016 to Dec 2017. Exome sequencing was performed for all patients. Variants in reported PPHN/pulmonary arterial hypertension (PAH)-related genes were assessed. Single nucleotide polymorphism (SNP) association and gene-level analyses were carried out in 74 PPHN cases and 115 non-PPHN controls with matched baseline characteristics.

**Results:**

Among the patient cohort, 74 (64.3%) patients were late preterm and term infants (≥ 34 weeks gestation) and 41 (35.7%) were preterm infants (< 34 weeks gestation). Preterm infants with PPHN exhibited low birth weight and a high frequency of bronchopulmonary dysplasia, respiratory distress syndrome (RDS) and mortality. Nine patients (only one preterm infant) were identified as harboring genetic variants, including three with pathogenic/likely pathogenic variants in *TBX4* and *BMPR2* and six with variants of unknown significance in *BMPR2*, *SMAD9*, *TGFB1*, *KCNA5* and *TRPC6*. Three SNPs (rs192759073, rs1047883 and rs2229589) in *CPS1* and one SNP (rs1044008) in *NOTCH3* were significantly associated with PPHN (*p* < 0.05). *CPS1* and *SMAD9* were identified as risk genes for PPHN (*p* < 0.05).

**Conclusions:**

In this study, we identified genetic variants in PPHN patients, and we reported *CPS1*, *NOTCH3* and *SMAD9* as risk genes for late preterm and term PPHN in a single-center Chinese cohort. Our findings provide additional genetic evidence of the pathogenesis of PPHN and new insight into potential strategies for disease treatment.

**Electronic supplementary material:**

The online version of this article (10.1186/s12931-019-1148-1) contains supplementary material, which is available to authorized users.

## Background

Persistent pulmonary hypertension of the newborn (PPHN) is caused by a failure in the normal circulatory transition at birth and is characterized by elevated pulmonary vascular resistance (PVR), which leads to right-to-left shunting and hypoxemia. The incidence of PPHN ranges from 2 to 6 per 1000 live births and the mortality rate is 10–20% [1]. PPHN can be idiopathic or may be caused by multiple pulmonary diseases including perinatal asphyxia, meconium aspiration syndrome (MAS), respiratory distress syndrome (RDS), pulmonary dysplasia and congenital diaphragmatic hernia [2].

To date, only a few genetic polymorphisms have been identified in infants with PPHN. Pearson et al. first found that a T1405 N variant of carbamoyl phosphate synthetase I (*CPS1*) exhibited a different distribution between infants with PPHN and the general population [3]. A homozygous missense variant (L326R) in the *ABCA3* gene was identified in a newborn with severe hypoxemic respiratory failure and refractory pulmonary hypertension [4]. Single nucleotide polymorphisms (SNPs) in the corticotropin-releasing hormone receptor 1 (*CRHR1*) and corticotropin-releasing hormone-binding protein (*CRHBP*) genes were significantly associated with PPHN [5]. Most recently, rs2070699 in endothelin 1 (*EDN1*) was found to increase the risk of PPHN with respiratory distress [6].

PPHN is a subgroup of pulmonary arterial hypertension (PAH), which is a complex disorder characterized by elevation of PVR and failure of right heart function. It is a common complication of many clinical diseases. PAH is classified into five types according to the pathogenesis of the disease (WHO classification) and PPHN is a specific group [7]. Pathogenic variants among several genes have been reported in PAH patients exhibiting both adulthood onset and childhood onset. Bone morphogenic protein receptor type 2 (*BMPR2*), a member of the transforming growth factor beta (TGF-β) superfamily, is associated with 70% of familial pulmonary arterial hypertension (FPAH)/heritable pulmonary arterial hypertension (HPAH) cases and 20% of idiopathic pulmonary hypertension (IPAH) cases [8]. *SMAD9* [9], *CAV1* [10], *KCNK3* [11] are also known PAH genes listed in the Online Mendelian Inheritance in Man (OMIM) database. Hereditary hemorrhagic telangiectasia (HHT) gene variants in the activin receptor-like kinase 1 (*ACVRL1)* [12] and endoglin (*ENG*) [13] have been identified in PAH patients. To date, no causal genes for PPHN have been reported, and the genetic etiology remains unclear. We suggest that the genetic pathogenesis of PPHN may share some similarities with PAH in adults and children.

Therefore, in the present study, we applied clinical exome sequencing to investigate the genetic etiology of PPHN in 115 Chinese patients. We aimed to identify causal variants in reported PPHN/PAH-related genes and genetic risk polymorphisms for PPHN patients.

## Methods

### Study participants

In this study, neonates who were admitted to the neonatal intensive care unit (NICU) of the Children’s Hospital of Fudan University from January 2016 to December 2017 were recruited. The inclusion criteria were neonates with hypoxemic respiratory failure with a clinical diagnosis of pulmonary hypertension within 28 days after birth. Neonates with PPHN were diagnosed based on clinical and echocardiographic data. The criteria used for diagnosis was from previously published work by Alano et al. [14]: 1) the clinical criteria included a preductal/postductal oxygen saturation difference of > 10%, and 2) the echocardiographic criteria included a structurally normal heart and elevated pulmonary artery pressure (PAP). The last criterion was considered to be present if there was either right-to-left or bidirectional flow across the patent ductus arteriosus or foramen ovale, or systolic pulmonary arterial pressure was greater than or equal to the systemic blood pressure according to Doppler measurement of the tricuspid-regurgitation jet. Neonates with congenital anomalies or structural congenital heart disease other than patent ductus arteriosus and patent foramen ovale were excluded.

Infants who did not have pulmonary hypertension were collected as non-PPHN controls. Late preterm and term PPHN cases and control infants with matched baseline characteristics were included for further case-control analysis. All guardians of the subjects included in this study were provided with appropriate informed consent. This study was approved by the ethics committee of the Children’s Hospital of Fudan University.

### Clinical exome sequencing

Genomic DNA was extracted from whole blood using the QIAamp DNA Blood Mini Kit (Qiagen, Hilden, Germany). The DNA concentration was measured using a Nano-Drop spectrophotometer (ND-1000, Thermo Fisher Scientific Inc., Waltham, MA, USA). The clinical exome panel used for sequencing, which covered 2742 genes causing inherited diseases, was generated using the Agilent ClearSeq Inherited Disease Kit (Agilent Technologies, Santa Clara, CA) and Illumina Cluster and SBS Kits (Illumina Inc., San Diego, CA, USA). Sequencing was performed on the Illumina HiSeq 2000/2500 platform (Illumina Inc., San Diego, CA, USA). Clean reads were aligned to the reference human genome (UCSC hg19) by the Burrows-Wheeler Aligner (BWA; v.0.5.9-r16). After quality control, variants were obtained using GATK.

### Variant annotation and classification

Variants were annotated by using ANNOVAR [15] and VEP [16] software, and the Human Gene Mutation Database (HGMD, professional version) and ClinVar database (https://www.ncbi.nlm.nih.gov/clinvar/). Missense variants were evaluated with SIFT [17], PolyPhen-2 [18] and MutationTaster [19]. Common variants with a minor allele frequency (MAF) > 0.01 were excluded based on the Exome Aggregation Consortium (ExAC) database (http://exac.broadinstitute.org/), 1000 Genomes database (http://www.internationalgenome.org/) and our in-house database. Synonymous and intronic variants falling outside the +/− 15 bp boundaries of exons were also discarded. MAFs for all variants were collected from the ExAC database and the Genome Aggregation Database (GnomAD, http://gnomad.broadinstitute.org/). Causal variants were further selected in 25 reported PPHN/PAH disease-related genes (Additional file 1: Table S1). Only variants with a very low frequency (MAF < 0.005) in both the overall and East Asian populations and were absent from infants without PPHN in our in-house database (including 24,336 samples) were further considered to be pathogenic. The pathogenicity of the variants was defined based on the American College of Medical Genetics and Genomics (ACMG) criteria [20]. Specifically, a case was classified as molecularly diagnosed when the identified pathogenic or likely pathogenic (P/LP) variants were truncating variants or reported missense variants detected in a disease gene that sufficiently explained the phenotypes of the studied individual. Variants with unknown significance (VUSs) were associated with one or more clinical phenotypes of patients and were absent or present in the GnomAD/ExAC databases with low frequencies.

### SNP calling and quality control

**Fig. 1:**
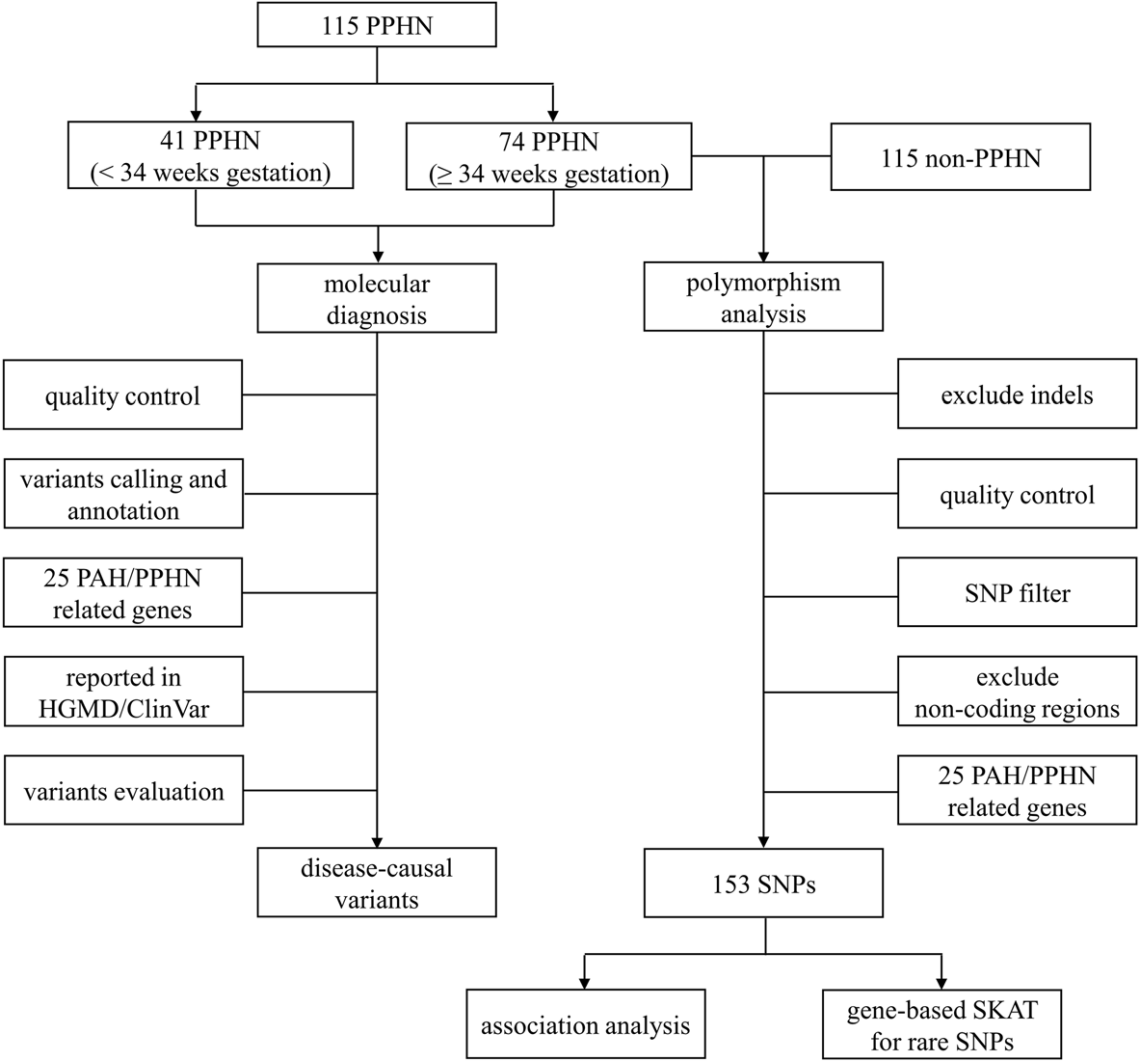
Flow diagram of the genetic testing and analysis strategy applied in the study. Exome sequencing was performed for all patients, and sequencing data were used for the following analysis. Disease-causing variants among 25 PPHN/PAH-related genes were analyzed in 115 PPHN cases. SNP association analysis and gene-level analysis were carried out in 74 PPHN cases and 115 non-PPHN controls

Only SNPs with high confidence (depth ≥ 10, call ratio ≥ 0.2 for heterogeneous variants and ≥ 0.6 for homogeneous variants) that were present in at least one individual were selected for further analysis. The following filtering criteria were used to filter SNPs for case-control analysis: 1) missing rate < 0.2, 2) MAF (PLINK) > 0.001; 3) control Hardy-Weinberg equilibrium (HWE) (PLINK) > 0.001. SNPs within the sequencing coding region of 25 PPHN/PAH-related genes were included for further analysis. The workflow is shown in Fig. 1.

### Statistical analysis

Differences in clinical characteristics in different study groups were analyzed using the t-test for continuous variables and the chi-square test for categorical variables. A two-sided type I error of 0.05 was used to test for statistical significance. The statistical analyses were performed with SPSS version 16 (SPSS Inc., Chicago, IL, USA). The results of SNP association analysis using exome sequence data were analyzed with the chi-square test using PLINK software (http://zzz.bwh.harvard.edu/plink/, version 1.07). Gene-level analysis using the Sequence Kernel Association test (SKAT) [21] in the R package with default settings was carried out for rare SNPs (MAF < 0.05).

## Results

### Patient characteristics

**Fig. 2:**
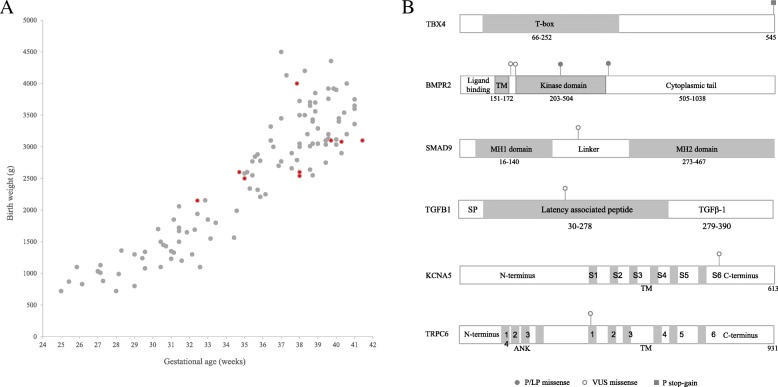
Gestational age and birth weight of 115 PPHN patients and schematic representation of functional domains and locations of variants in the 6 PAH disease gene proteins. **a** Birth weight (g) was plotted on the y axis and gestational age was plotted on the x axis. One dot represents one patient. Red dots were patients identified with genetic variants. **b** The figure shows variants in the TBX4, BMPR2, SMAD9, TGFB1, KCNA5 and TRPC6 proteins. Missense pathogenic/likely pathogenic (P/LP) variants are indicated with solid circles; missense variants with unknown significance (VUSs) are indicated with hollow circles; and one pathogenic stop-gain variant is indicated with a square. TM: transmembrane region; SP: signal peptide; ANK: ankyrin

A total of 115 infants diagnosed with PPHN were enrolled in this study. The average gestational age was 34.9 weeks, and the average birth weight was 2516.2 g. The majority of the patients were male (64, 55.7%). The age at diagnosis for all patients ranged from 1 day to 5 days after birth, and the majority (110 patients) were diagnosed within 3 days. RDS (53, 46.1%) and pneumonia (44, 38.3%) were two major primary diagnoses in PPHN cases. Among all cases, 17 were treated with inhaled nitric oxide (iNO), and 4 were treated with extracorporeal membrane oxygenation (ECMO). The patients’ demographic and clinical characteristics are shown in Table 1 and Fig. 2a.

**Table 1:** Demographic and clinical characteristics of study population

Characteristics	All PPHN (*n* = 115)	Preterm (< 34 weeks) PPHN (*n* = 41)	Late preterm and term (≥34 weeks) PPHN (*n* = 74)	*P* value (< 34 weeks PPHN vs ≥34 weeks PPHN)	Non-PPHN (*n* = 115)	*P* value (≥34 weeks PPHN vs non-PPHN)
Baseline characteristics						
Male gender, n(%)	64 (55.7)	24 (58.5)	40 (54.1)	0.6430	62 (53.9)	0.9849
Birth weight (g), mean(±SD)	2516.2 ± 1006.2	1358.8 ± 391.7	3157.4 ± 572.6	< 0.001	3169.3 ± 576.6	0.8896
Gestational age (weeks), mean(±SD)	34.9 ± 4.4	29.7 ± 2.3	37.8 ± 1.9	< 0.001	38.2 ± 1.4	0.1649
Premature (< 37 weeks), n(%)	60 (52.2)	41 (100)	19 (25.7)	< 0.001	17 (14.8)	0.0627
Caesarean, n(%)	76 (66.1)	24 (58.5)	52 (70.3)	0.2030	70 (60.9)	0.1873
Primary diagnosis for PPHN cases						
Respiratory distress syndrome, n(%)	53 (46.1)	27 (65.9)	26 (35.1)	0.0015	/	/
Meconium aspiration syndrome, n(%)	10 (8.7)	0 (0)	10 (13.5)	0.0135	/	/
Pneumothorax, n(%)	17 (14.8)	3 (7.3)	14 (18.9)	0.0932	/	/
Pneumonia, n(%)	44 (38.3)	14 (34.1)	30 (40.5)	0.4992	/	/
Bronchopulmonary dysplasia, n(%)	10 (8.7)	9 (22.0)	1 (1.4)	0.0004	/	/
Treatment and prognosis for PPHN cases						
Ventilation time (days), mean(±SD)	12.7 ± 18.9	22.2 ± 26.9	6.8 ± 6.7	0.0015	/	/
iNO, n(%)	17 (14.8)	1 (2.4)	16 (21.6)	0.0055	/	/
Vasoactive agents therapy, n(%)	64 (55.7)	19 (46.3)	45 (60.8)	0.1347	/	/
ECMO, n(%)	4 (3.5)	0 (0)	4 (5.4)	0.2952	/	/
Mortality, n(%)	14 (12.2)	7 (17.1)	7 (9.5)	0.2474	/	/

*iNO* Inhaled nitric oxide, *ECMO* Extracorporeal membrane oxygenation

PPHN occurred in most (64.3%, 74/115) of the late preterm and term infants (≥ 34 weeks gestation), and 35.7% (41/115) of the patients were preterm infants (< 34 weeks gestation). Preterm infants exhibited lower birth weight, a higher incidence of RDS and were more likely to require longer ventilation treatment. iNO treatment was usually carried out in late preterm and term infants, and ECMO was only performed in late preterm and term infants. Specifically, 17 infants (16 infants > 34 weeks gestation and 1 infant < 34 weeks gestation) were treated with iNO, and 4 infants (all > 34 weeks gestation) were treated with ECMO. Mortality was higher in preterm patients than late preterm and term infants (17.1% vs 9.5%).

### Sequencing results

An average of 27.5 million effective reads were generated with an average sequencing depth of 230.98-fold per target in 115 PPHN patients and 115 non-PPHN controls. In total, 99.8% of the target region was covered, among which 99.6% was covered at least 10-fold, and 99.2% was covered at least 20-fold.

### Genetic variant identification

#### PPHN/PAH-related genes

In total, 9 phenotype related variants spanning 6 PAH-related genes were identified in 9 patients (7.8%). The variants included 3 P/LP variants and 6 VUSs, among which two were reported disease-causing variants, and seven were novel. The 3 P/LP variants, including one stop-gain variant (c.1633G > T. p.G545X) in *TBX4* and two reported PAH-associated missense variants (c.1535A > C, p.K512 T [22]; and c.1094G > A, p.R365H) [23] in *BMPR2*, were identified in three patients. K512 T was reported in one adult patient of western European origin with an age at onset of 19–50 years, and R365H was reported in one adult IPAH patient in Spain. Two novel missense variants (c.565A > G, p.M189 V; and c.596A > T, p.D199V) in *BMPR2* and one novel missense variant (c.587C > T, p.A196V) in *SMAD9* were classified as VUSs. In addition, three VUSs in PAH genes (*TGFB1* (c.426A > C, p.E142D), *KCNA5* (c.1645G > T, p.D549Y) and *TRPC6* (c.1327 T > A, p.F443I)) were also found individually in three patients. Detailed information on all variants is shown in Table 2 and Fig. 2b.

**Table 2:** Genetic variants identified in 12 patients

Patient	Gene	Disease (OMIM)	Inheritance pattern	Position	RefSeq	Nucleotide Change	Protein Change	Zygosity	Variant type	GnomAD/ExAC	Prediction (SIFT/polyphen2/MutationTaster)	Previously reported	Classification
PPHN/PAH related genes													
P001	*TBX4*	Ischiocoxopodopatellar syndrome, [MIM:147891]	AD	chr17:59560872	NM_018488	c.1633G > T	p.G545X	Het	stop-gain	./.	././D	/	LP
P002	*BMPR2*	Pulmonary hypertension, familial primary, 1, with or without HHT, [MIM:178600]; Pulmonary hypertension, primary, fenfluramine or dexfenfluramine-associated, [MIM:178600]	AD	chr2:203417560	NM_001204	c.1535A > C	p.K512 T	Het	missense	./.	T/B/D	PMID: 11115378	P
P003	*BMPR2*			chr2:203395643	NM_001204	c.1094G > A	p.R365H	Het	missense	16/2 het carriers (0 in EAS)	T/B/D	PMID: 27453251	LP
P004	*BMPR2*			chr2:203379646	NM_001204	c.565A > G	p.M189 V	Het	missense	./.	T/B/D	/	VUS
P005	*BMPR2*			chr2:203379677	NM_001204	c.596A > T	p.D199V	Het	missense	.	D/D/D	/	VUS
P006	*SMAD9*	Pulmonary hypertension, primary, 2, [MIM:615342]	AD	chr13:37446878	NM_001127217	c.587C > T	p.A196V	Het	missense	5/2 het carriers (0 in EAS)	T/B/N	/	VUS
P007	*TGFB1*	Camurati-Engelmann disease, [MIM:131300]	AD	chr19:41854290	NM_000660	c.426A > C	p.E142D	Het	missense	./.	T/B/D	/	VUS
P008	*KCNA5*	Atrial fibrillation, familial, 7, [MIM:612240]	AD	chr12:5154958	NM_002234	c.1645G > T	p.D549Y	Het	missense	./.	D/B/N	/	VUS
P009	*TRPC6*	Glomerulosclerosis, focal segmental, 2, [MIM:603965]	AD	chr11:101353863	NM_004621	c.1327 T > A	p.F443I	Het	missense	./.	D/D/D	/	VUS
PPHN/PAH unrelated genes													
P010	*ABCC8*	Hyperinsulinemic hypoglycemia, familial, 1, [MIM:256450]; Hypoglycemia of infancy, leucine-sensitive, [MIM:240800]	AD	chr11:17436118	NM_000352	c.2331G > A	p.W777X	Het	stop-gain	./.	T/./A	/	P
P011	*OPLAH*	5-oxoprolinase deficiency, [MIM:260005]	AD/AR	chr8:145106241	NM_017570	c.3853C > T	p.Q1285X	Het	stop-gain	18/6 het carriers (all in EAS)	D/./.	/	LP
P011	*OPLAH*			chr8:145107351	NM_017570	c.3303 + 1G > C	.	Het	splicing	./.	././.	/	LP
P012	*KCNH2*	Long QT syndrome 2, [MIM:613688]; Short QT syndrome 1, [MIM:609620]	AD	chr7:150654411	NM_000238	c.1096C > T	p.R366X	Het	stop-gain	./.	T/./A	PMID: 11468227	P

*AD* Autosomal dominant, *AR* Autosomal recessive, *het* Heterogeneous, *SIFT-D* Deleterious, *SIFT-T* Tolerated, *polyphen2-B* Benign, *polyphen2-D* Porobably damaging, *polyphen2-P* Possibly damaging, *MutationTaster-D* Disease_causing: probably deleterious, *MutationTaster-N* Polymorphism: probably harmless, *EAS* East Asian, *LP* Likely pathogenic, *P* Pathogenic, *VUS* Variants with unknown significance

#### PPHN/PAH-unrelated genes

In three patients who presented with other clinical features, P/LP variants were identified in PPHN/PAH-unrelated genes. Two patients exhibited metabolic diseases: a boy with hypoglycemia carried a novel stop-gain variant (c.2331G > A, p.W777X) in *ABCC8* that was associated with hypoglycemia in infancy [MIM:240800], and a girl with metabolic acidosis carried two LP variants, including one stop-gain variant (c.3853C > T, p.Q1285X) and one splicing variant (c.3303 + 1G > C) in *OPLAH*, that were associated with 5-oxoprolinase deficiency [MIM:260005]. The other patient was a female with multiple arrhythmias, in whom a reported stop-gain variant (c.1096C > T, p.R366X) in *KCNH2* related to long QT syndrome 2 [MIM:613688] and short QT syndrome 1 [MIM:609620] was identified.

### Clinical phenotypes in nine patients with PPHN/PAH-related genes

Among the reported PPHN/PAH-related genes, *TBX4* and *BMPR2* were two major genetic factors in PPHN. Patients P001-P003 carried P/LP variants in these two genes. P001 was a girl with a stop-gain variant in *TBX4* who presented with severe PPHN with a maximal oxygenation index (MOI) value of 27. She was treated with ventilation for 8 days and vasoactive agent therapy for 5 days. Patient P002 displayed a moderate phenotype with a PAP of 50 mmHg and an MOI value of 12.5. She was treated with ventilation for 8 days, sildenafil therapy for 5 days and vasoactive agent therapy for 9 days. This girl was identified as harboring a pathogenic missense variant (K512 T) in *BMPR2*, located at the cytoplasmic tail of the protein. Patient P003 was a girl who exhibited a severe phenotype with a PAP of 82 mmHg and an MOI value of 22.9. She was treated with ventilation for 21 days, sildenafil therapy for 11 days and vasoactive agent therapy for 15 days. The patient had been administered ECMO treatment for 15 days. An R365H variant was identified in this patient. The residue is located in the kinase domain, which is important for kinase activity (Fig. 2b). This variant has been reported as a possibly pathogenic variant for PAH. Although this variant appears in GnomAD (16 heterogeneous carriers) and ExAC (2 heterogeneous carriers), it is absent in the East Asian population in these two databases and in our large internal database with 24,336 non-PPHN samples. Therefore, this variant was considered an LP for PPHN and needs to be further confirmed in the patients’ family.

Patients P004 and P005, who exhibited the novel VUSs M189 V and D199V in *BMPR2*, located between the transmembrane region and the kinase domain of the protein, were diagnosed with mild PPHN. Patients P006 and P008-P009, who harbored VUSs in *SMAD9*, *KCNA5* and *TRPC6*, also displayed moderate to mild phenotypes. A boy P007 who carried an E142D variant in *TGFB1* exhibited severe PPHN with an MOI value of 23.1. He was treated with ventilation for 11 days and vasoactive agent therapy for 7 days. This residue is located in the α3-helix of the ARM domain of the latency-associated peptide (LAP) region (Fig. 2b). LAP is required for homodimer assembly and protein secretion, and regulates the bioactivity of TGF-β [24]. Detailed information on the clinical phenotypes is shown in Additional file 2: Table S2.

### Risk polymorphism identification

We included 74 PPHN cases and 115 non-PPHN controls with matched clinical characteristics (≥ 34 weeks gestational age) in case-control analysis to exclude the influence of nongenetic risk factors. All baseline characteristics of the case and control groups were similar (Table 1). After quality control and SNP filtering, 153 SNPs remained in 25 PPHN/PAH-related genes. Three SNPs in *CPS1* and one SNP in *NOTCH3* were significantly associated with PPHN (Table 3). The most significant SNPs were rs192759073 in *CPS1* and rs1044008 in *NOTCH3* (*p* = 0.03). The other two SNPs in *CPS1*, rs1047883 and rs2229589 were in linkage disequilibrium (LD, *r*^2^ = 1) and exhibited a relatively high frequency in public databases (0.45). We considered the first two SNPs (rs192759073 in *CPS1* and rs1044008 in *NOTCH3*) to be better risk markers for PPHN. Gene-level analysis was performed for 128 rare SNPs (MAF < 0.05) spanning 15 genes. Among these gene sets, *CPS1* was associated with PPHN at *p* = 0.006, which was consistent with the association of *CPS1* SNPs and PPHN (Additional file 3: Table S3). *SMAD9* was also associated with PPHN at *p* = 0.039.

**Table 3:** Significant SNPs in PAH related genes with PPHN

SNP	Position	Gene	Alleles	MAF in cases	MAF in controls	*P* value	Frequency in GnomAD_EAS	Frequency in ExAC_EAS
rs192759073	chr2:211438090	*CPS1*	C > T	0.02027	0	0.030	0.007321	0.006702
rs1044008	chr19:15272001	*NOTCH3*	C > T	0.02027	0	0.030	0.001627	0
rs1047883	chr2:211456637	*CPS1*	G > A	0.5338	0.4261	0.041	0.4515	0.4534
rs2229589	chr2:211456639	*CPS1*	T > C	0.5338	0.4261	0.041	0.4515	0.4534

SNP rs1047883 is in LD with rs2229589 (*r*^2^ = 1). *MAF* Minor allele frequency, *EAS* East Asian

## Discussion

PPHN is a severe clinical problem and accounts for ~ 6% of our NICU patients. The role of genetics in the pathogenesis of PPHN remains elusive. The present study investigated the genetic contributions to the pathogenesis of PPHN in 115 Chinese PPHN patients using exome sequencing. Among all cases, 41 were preterm infants and 74 were late preterm and term infants. We identified three patients with P/LP variants in *TBX4* and *BMPR2* and six patients with VUSs in *BMPR2* and 4 other reported PAH-related genes. *CPS1*, *NOTCH3* and *SMAD9* were identified as important risk genes for late preterm and term PPHN through case-control analysis.

PPHN has generally been recognized to occur among late preterm and term infants, but studies have reported an increasing rate of PPHN in preterm infants [25]. In this study, most of the infants with PPHN were late preterm and term infants (≥ 34 weeks gestation), and preterm infants also accounted for 35.7% of the patients. Among the 9 patients with genetic findings, only 1 patient with c.596A > T (p.D199V) in *BMPR2* was born before 34 weeks gestation (32 + 3 weeks). The genetic diagnosis rates were different in the two groups (8/74 in the ≥34-week gestation group vs 1/41 in the < 34-week gestation group). Our findings indicated that preterm complications play major roles in preterm infants with PPHN, while genetic factors have a greater effect on late preterm and term infants.

In terms of the genetic background of PPHN, previous studies have not found the disease-causing gene for PPHN patients thus far, and only polymorphisms in 5 genes have been reported to be associated with PPHN. PAH has been widely studied in both adults and children, and 20 genes have been associated with the development of the disease (Additional file 1: Table S1). The genetic etiology of PPHN in newborns is complex and unclear and may share some similarities with PAH in adults and children. In this study, we identified several variants in PAH-related genes, which verified that PAH and PPHN potentially exhibit a common genetic pathogenesis. We also found three disease-causing genes in the three other patients. However, these genes were not associated with the development of pulmonary hypertension. Further studies are needed to investigate other potential disease-causing genes related to PPHN.

Among the genes identified in this study, several genetic variants in the BMP/TGF–β/SMAD pathways were identified, including three P/LP variants in *TBX4* and *BMPR2* and one VUS in *TGFB1* related to severe clinical phenotypes in four patients. BMP/TGF–β/SMAD signaling (especially *BMPR2*) has been reported to be involved in the regulation of the proliferation and apoptosis of pulmonary arterial smooth muscle cells (PASMCs) and pulmonary arterial endothelial cells (PAECs) [26]. In a previous study of PAH, *BMPR2* variants were more commonly found in females than males (3.6:1 ratio in adult-onset PAH cases and 1.7:1 ratio in pediatric-onset PAH cases) [27]. The sex ratio was similar (3:1, female:male ratio) in our study among the 4 *BMPR2* variant-carrying PPHN patients. *TBX4* is a member of the T-box genes that is important for the development of airway branching and the regulation of lung fibrosis. *TBX4* variants have been reported in childhood-onset PAH patients [28] and might contribute to PAH by decreasing the activation of the BMP/TGF–β/SMAD signaling pathways [29]. *TGFB1* (transforming growth factor β1) is a member of the TGF-β superfamily, whose members are important modulators of cell growth, inflammation and apoptosis. *TGFB1* can suppress the proliferation and migration of endothelial and smooth muscle cells and thereby inhibit vascular remodeling. Variants in *TGFB1* might affect its function and lead to pulmonary hypertension [30]. Both the TGF-β and BMP signaling pathways ultimately converge on SMADs. One rare *SMAD9* variant, A196V, located in the linker domain of the protein was identified in one patient (Fig. 2b). The linker region of SMAD9 is rendered shorter than those of other SMADs, which suppresses its transcriptional activity and ability to activate BMP signaling, while facilitating interaction with other molecules [31]. In addition, two ion channel genes, which might also play important roles in PAH, were identified in our patients. The Kv1.5 channel gene (*KCNA5*) is a pore-forming α-subunit that forms a voltage-gated K^+^ channel in PASMCs. Downregulation of *KCNA5* causes membrane depolarization and increases the cytosolic Ca^2+^ concentration, resulting in pulmonary vasoconstriction, and pulmonary vascular remodeling [32]. A novel D549Y variant in *KCNA5* was identified in one girl. The residue is located in the C-linker region following transmembrane domain segment 6. Another novel variant, F443I in *TRPC6*, was identified in another patient. *TRPC6* is an important member of the TRPC channels of the transient receptor potential (TRP) superfamily expressed in the lungs and PASMCs [33]. A SNP in the promoter region of *TRPC6* has been demonstrated to increase the risk of IPAH by recruiting NF-κB [34].

Furthermore, we performed SNP association and gene-level analyses in 25 PPHN/PAH-disease related genes among 74 late preterm and term PPHN cases and 115 controls with matched clinical characteristics to further investigate the genetic etiology of PPHN. We identified 3 SNPs in *CPS1* and 1 SNP in *NOTCH3* associated with PPHN. The *CPS1* SNPs rs192759073 and rs2229589 are synonymous variants, and rs1047883 is a missense variant. The heterozygous rs192759073 T allele was identified in 3 female PPHN patients and none of the controls. For rs1047883 and rs2229589, homozygous SNPs were found in 19 PPHN cases and 20 controls and heterozygous SNPs were found in 41 PPHN cases and 58 controls. The synonymous SNP rs1044008 in *NOTCH3* was detected in three PPHN patients (heterozygous). These SNPs are reported to be associated with PPHN for the first time in this study. *CPS1* (carbamoyl phosphate synthase 1) encodes one of the key enzymes located in mitochondria involved in the urea cycle. A functional deficiency in the CPS1 enzyme can affect the catalysis of the first step of the urea cycle and the generation of nitric oxide, which plays a critical role in regulating pulmonary vascular resistance [35]. Sixteen polymorphisms, including three in coding regions (rs1047891, rs2287599 and rs41272667) of *CPS1* [3, 36], have been reported to be associated with PPHN. However, rs1047891 and rs2287599 were not significant in our cohort, and rs41272667 (close to the noncoding region) was not included in our study. The reason for this difference may be that the genetic risk factors for PPHN differ in different ethnic populations. *NOTCH3* belongs to the Notch signaling pathway, which plays an important role in the regulation of cellular proliferation, differentiation and apoptosis. Heterozygous variants in *NOTCH3* might affect cell proliferation and NOTCH3-HES5 signaling resulting in PAH [37]. Gene-level analysis also identified *CPS1* and *SMAD9* as genetic risk factors for PPHN.

There are several limitations to our study. We used clinical exome sequencing (with 16 PPHN/PAH disease-related genes included in the panel) for genetic testing, and the other 9 genes need to be further studied. Genetic risk polymorphisms are usually identified in noncoding regions, which cannot be detected using exome sequencing panels. However, exome sequencing provides more information for variants spread throughout genes than candidate SNP genotyping has provided in previous studies. Additionally, we could not study the association between nitric oxide metabolites and PPHN since the plasma concentrations of nitric oxide metabolites were not measured/recorded for all patients.

## Conclusions

In summary, this study identified 9 rare variants in PAH-related genes (*TBX4*, *BMPR2*, *SMAD9*, *TGFB1*, *KCNA5* and *TRPC6*) in 9 PPHN patients in a Chinese cohort. We also revealed an association between the *CPS1*, *NOTCH3* and *SMAD9* genes and late preterm and term PPHN infants. Our findings have important clinical implications for the earlier detection of PPHN in preterm infants and provide more insight into the pathogenesis of PPHN and potential strategies for disease treatment.

## Additional files

Additional file 1: **Table S1** Reported PAH/PPHN related genes. (DOCX 18 kb)

Additional file 2: **Table S2** Clinical phenotypes of 9 genetic positive PPHN patients. (DOCX 17 kb)

Additional file 3: **Table S3** Gene-level analysis for rare variants. (DOCX 15 kb)

## Data Availability

The datasets used and/or analyzed during the current study are available from the corresponding author upon reasonable request.

## Abbreviations

ACMG: American College of Medical Genetics and Genomics
ECMO: Extracorporeal membrane oxygenation
ExAC: Exome Aggregation Consortium
GnomAD: Genome Aggregation Database
HGMD: Human Gene Mutation Database
iNO: Inhaled nitric oxide
MAF: Minor allele frequency
MAS: Meconium aspiration syndrome
MOI: Maximal oxygenation index
NICU: Neonatal intensive care unit
OMIM: Online Mendelian Inheritance in Man
P/LP variants: Pathogenic or likely pathogenic variants
PAH: Pulmonary arterial hypertension
PAP: Pulmonary artery pressure
PPHN: Persistent pulmonary hypertension of the newborn
PVR: Pulmonary vascular resistance
RDS: Respiratory distress syndrome
SNP: Single nucleotide polymorphism
VUSs: Variants with unknown significance
