# Supplementary material for: Identification of genetic factors underlying persistent pulmonary hypertension of newborns in a cohort of Chinese neonates
Source: Respir Res. 2019 Aug 5;20:174. doi: 10.1186/s12931-019-1148-1 (PMC6683566; doi:10.1186/s12931-019-1148-1)
Supplement: Supplementary file 1 — Table S1 Reported PAH/PPHN related genes. (DOCX 18 kb) [file 12931_2019_1148_MOESM1_ESM.docx]

| **Table S1. Reported PAH/PPHN related genes.** | | | | |
| --- | --- | --- | --- | --- |
| **Gene** | **Disease** | **PMID (year)** | **OMIM-PAH related gene** | **Included in the panel sequencing** |
| *BMPR2* | FPAH;PAH;IPAH/HPAH(children) | 10973254(2000);18159113(2008);22632830(2012) | Yes | Yes |
| *ACVRL1(AK1)* | PAH;IPAH/HPAH(children) | 14684682(2004); 18159113(2008);22632830(2012) |  | Yes |
| *ENG* | PAH | 15115879(2004) |  | Yes |
| *SMAD9* | IPAH(children);PAH | 19211612(2009);21898662(2011) | Yes | Yes |
| *SMAD1* | PAH | 21898662(2011) |  | No |
| *SMAD4* | PAH | 21898662(2011) |  | Yes |
| *CAV1* | HPAH/IPAH | 22474227(2012) | Yes | Yes |
| *BMPR1B(ALK6)* | IPAH(children) | 22374147(2012) |  | Yes |
| *KCNK3* | FPAH/IPAH;PAH | 23883380(2013);27649371(2017) | Yes | No |
| *TBX4* | IPAH/HPAH(children) | 23592887(2013) |  | Yes |
| *EIF2AK4(GCN2)* | Pulmonary veno-occlusive disease (PVOD) | 24292273(2014) |  | No |
| *TGFB1* | IPAH | 18097622(2008) |  | Yes |
| *TRPC6* | IPAH | 19380626(2009) |  | Yes |
| *KCNA5* | IPAH | 17267549(2007) |  | Yes |
| *THBS1* | FPAH | 22198906(2012) |  | No |
| *NOTCH3* | PAH(children) | 24936512(2014) |  | Yes |
| *TOPBP1* | IPAH | 24702692(2014) |  | No |
| *GDF2(BMP9)* | PAH(children) | 26801773(2016) |  | No |
| *EDNRA* | PAH | 21773759(2012) |  | Yes |
| *PTGIS* | PAH | 24605778(2014) |  | Yes |
| *CPS1* | PPHN | 11407344(2001);28609431(2017) | Yes (susceptibility to) | Yes |
| *CRHR1* | PPHN | 22258127(2012) |  | No |
| *CRHBP* | PPHN | 22258127(2012) |  | No |
| *ABCA3* | PPHN | 17719949(2007) |  | Yes |
| *EDN1* | PPHN | 27425626(2016) |  | No |
| HPAH: hereditary PAH; IPAH: idiopathic PAH; FPAH: familial PAH; OMIM: Online Mendelian Inheritance in Man. | | | | |
