# Supplementary material for: Identification of genetic factors underlying persistent pulmonary hypertension of newborns in a cohort of Chinese neonates
Source: Respir Res. 2019 Aug 5;20:174. doi: 10.1186/s12931-019-1148-1 (PMC6683566; doi:10.1186/s12931-019-1148-1)
Supplement: Supplementary file 2 — Table S2 Clinical phenotypes of 9 genetic positive PPHN patients. (DOCX 17 kb) [file 12931_2019_1148_MOESM2_ESM.docx]

| **Table S2. Clinical phenotypes of 9 genetic positive PPHN patients.** | | | | | | | | | | | | |
| --- | --- | --- | --- | --- | --- | --- | --- | --- | --- | --- | --- | --- |
| **Patient** | **Gender** | **Gestational age (week)** | **Birth weight (g)** | **Delivery** | **Primary diagnosis** | **PAP (mmHg)** | **MOI** | **Ventilation time (day)** | **iNO (day)** | **Sildenafil (day)** | **Vasoactive agent therapy (day)** | **ECMO (day)** |
| P001 | female | 38 | 2600 | cesarean section | MAS | 33 | 27 | 8 | / | / | 5 | / |
| P002 | female | 38 | 2540 | natural labor | RDS | 50 | 12.5 | 8 | / | 5 | 9 | / |
| P003 | female | 37+6 | 4000 | cesarean section | RDS | 82 | 22.9 | 21 | / | 11 | 15 | 15 |
| P004 | female | 40+2 | 3080 | cesarean section | MAS | 48 | 11.5 | 5 | / | 5 | 4 | / |
| P005 | male | 32+3 | 2150 | cesarean section | RDS | 35 | 3.9 | 5 | / | / | / | / |
| P006 | male | 35 | 2500 | cesarean section | RDS | 45 | 13 | 4 | / | / | / | / |
| P007 | male | 41+3 | 3100 | natural labor | Pneumonia | 83 | 23.1 | 11 | / | / | 7 | / |
| P008 | female | 34+5 | 2600 | natural labor | MAS, RDS | 36 | 6.1 | 3 | / | / | / | / |
| P009 | male | 39+5 | 3100 | cesarean section | Pneumonia | 34 | 7.2 | 5 | / | / | 5 | / |
| RDS: respiratory distress syndrome; MAS: meconium aspiration syndrome; PAP: pulmonary artery pressure; MOI: maximal oxygenation index; iNO: inhaled nitric oxide; ECMO: extracorporeal membrane oxygenation. | | | | | | | | | | | | |
