# Supplementary material for: Identification of genetic factors underlying persistent pulmonary hypertension of newborns in a cohort of Chinese neonates
Source: Respir Res. 2019 Aug 5;20:174. doi: 10.1186/s12931-019-1148-1 (PMC6683566; doi:10.1186/s12931-019-1148-1)
Supplement: Supplementary file 3 — Table S3 Gene-level analysis for rare variants. (DOCX 15 kb) [file 12931_2019_1148_MOESM3_ESM.docx]

| **Table S3. Gene-level analysis for rare variants.** | | |
| --- | --- | --- |
| **Chromosome** | **Gene** | ***p* value** |
| 2 | *CPS1* | 0.0064 |
| 13 | *SMAD9* | 0.0394 |
| 2 | *BMPR2* | 0.1553 |
| 19 | *NOTCH3* | 0.3077 |
| 4 | *BMPR1B* | 0.3897 |
| 9 | *ENG* | 0.4589 |
| 17 | *TBX4* | 0.4741 |
| 19 | *TGFB1* | 0.5000 |
| 12 | *KCNA5* | 0.5117 |
| 11 | *TRPC6* | 0.6226 |
| 16 | *ABCA3* | 0.6734 |
| 4 | *EDNRA* | 0.7030 |
| 7 | *CAV1* | 0.7990 |
| 20 | *PTGIS* | 0.8904 |
| 12 | *ACVRL1* | 0.9023 |
